# Acute Exposure to Bisphenol S Decreases In Vitro Right Atrial Contractility in Rats

**DOI:** 10.7759/cureus.51387

**Published:** 2023-12-31

**Authors:** Jayanti Pant, Radhika Agarwal, Srikant S, Latika Mohan

**Affiliations:** 1 Physiology, All India Institute of Medical Sciences, Rishikesh, Rishikesh, IND

**Keywords:** cgmp independent, no, atrial contractility, toxicity, bps

## Abstract

Aim/Objective: Bisphenols are widely used in the manufacturing of polycarbonate material and epoxy resins which constitute the essential component of plastic. Bisphenol A (BPA) has been reported to produce toxicity on organs in both animal and human studies. Therefore, plastic manufacturers are replacing BPA with other analogues that are considered to be safe, and BPA-free products are now available in the market. However, some studies have reported that bisphenol-S (BPS) also possesses toxic properties. It has been reported to depress ventricular contraction as well as produce ventricular arrhythmia on acute exposure. The present study was performed to examine the effect of BPS on in vitro spontaneously-beating right atria in rats.

Methods: In the present study, in vitro spontaneous contractions of right atria obtained from adult female rats of the Wistar strain were recorded. The atria were exposed to BPS (10^-6^-10 mM) and its effects on atrial contractions were recorded in the form of cumulative-concentration response with and without administration of antagonists namely atropine, L-NAME, and methylene blue.

Results: BPS decreased the rate as well as the force of atrial contractions. The changes produced in the rate and force of atrial contractions were not attributed to ethanol, which was used to prepare BPS solutions. The decrease in right atrial contractility produced by BPS was blocked by L-NAME; however, atropine and methylene blue were not able to antagonize the effects of BPS on atria.

Conclusions: The present study indicates the involvement of NO-dependent but cGMP independent pathway responsible for BPS‐induced cardio-toxicity

## Introduction

Bisphenol A (BPA) is widely used to manufacture polycarbonate material and epoxy resins to prepare plastic wares and inner linings of food and beverage cans. The major route of exposure to BPA is through the diet. Inhalation and absorption through the skin are other routes of exposure [1]. BPA can leach into food from the epoxy resin coatings of canned foods and consumer products such as polycarbonate tableware, food storage containers, water bottles, and baby bottles when they are exposed to high temperature, acidic pH, or when the plastic containers are washed by harsh detergents [2]. BPA has been reported to be secreted in breast milk and milk consumed through plastic bottles [3,4].

Exposure to BPA has been associated with numerous toxic effects like cancer, diabetes, disorders of reproduction, neurobehavioral alterations, and cardiovascular systems [5-10]. The chemical has been detected in the urine and blood of more than 90% of the United States population [11]. Due to the proven toxicity of BPA, nowadays, manufacturers are opting for other alternatives of BPA or BPA-free products. These alternatives are mostly analogues of BPA like bisphenol F, bisphenol AF, bisphenol S, etc. Of these, bisphenol S (BPS) is considered the most safe as it is very unlikely to leach out on exposure to heat. BPS is considered to be heat and sunlight-resistant due to the presence of two phenol groups on either side of the sulphonyl group [12].

The routes of exposure of humans to BPS are similar to BPA. BPS mainly enters our body by the oral route while consumption of canned foods and beverages. The other routes of its entry into our body are through inhalation and absorption from the skin while using currency notes and receipts of bills [13]. BPS has been reported to be present in shampoos, toothpaste, lotions, etc [14]. Further, studies have revealed that it is also present in maternal and cord serum, which indicates exposure to BPS through the placenta [15].

On ingestion, the liver metabolises BPS; 41% of BPS is glucuronidated by the liver and excreted through urine and the remaining BPS is available in the body. This is different from the metabolism of BPA, which is largely excreted through urine and only 0.50% is left back in the body. This is a matter of concern as this is expected to affect the tissues with chronic exposure to the chemical [16].

BPS has been reported to produce toxic effects similar to or even more than BPA [17]. BPS is reported to disrupt a cell’s normal functioning in extremely low doses. It has been found to affect neuronal development in zebrafish, neurobehavioral abnormalities in rodents, and reproductive toxicity in rodents [18,19]. Ventricular arrhythmias and depression of ventricular contractility are also reported on exposure to BPS in in vitro setups [20]. It is also found to be associated with obesity. Further, in humans, BPS has been detected in 81% of the urine samples, with a mean urinary concentration of 0.65 ng/mL (2.6 nM) [21]. Since BPS is presently the chemical of choice and is used enormously by plastic manufacturers, the safety of the chemical is doubtful according to very few studies on its effects on the heart. An earlier study by Ferguson et al. has shown the depressive effect of BPS on ventricular contractions and, therefore, it was hypothesized that BPS might have toxic effects on atrial contractility as well [20]. Hence, the present study was conducted to examine the effects of BPS on rat’s right atrial contractility and also to delineate the underlying mechanisms responsible for the same.

## Materials and methods

This study was conducted at the All India Institute of Medical Sciences, Rishikesh, India. The experiments were performed after obtaining approval from the Institutional Animal Ethics Committee (approval number: IAEC/AIIMS/Rish/Psy/12/20). Adult female albino rats of the Wistar strain weighing 150-200 g were used in the present study. Female rats were preferred in the present study since BPS has been reported to act on myocytes by oestrogen receptor β signalling [22 ]. The rats were kept in a room in the animal house of the institute taking precautions for proper maintenance of temperature, humidity and light with an ad libitum* s*upply of food and water as per the institute's guidelines.

Dissection and recording

The dissection was performed as per the method reported elsewhere [23]. Initially, the rats were sacrificed by cervical dislocation followed by exsanguination. This was followed by the removal of the skin of the thorax region followed by cutting the thorax. Thereafter, the heart was dissected and immersed in chilled Krebs Ringer solution; 100% O_2 _was used to bubble the solution. The right atrium was dissected out carefully and one end of the atrium was tied with thread to the tissue holder. The opposite end of the atrium was tied to a thread, which was used to fasten to a transducer. The tissue was placed in an organ bath, which was filled with Krebs Ringer solution. The temperature of the organ bath was maintained at 28 ± 1°C. The opposite end of the atrium was then secured to force displacement transducer. This preparation was allowed to stabilize for 30 min. After stabilization, the spontaneous atrial contractions were recorded by a physiograph (Recorders & Medicare Systems P Ltd (RMSIndia), Haryana, India).

Drugs and solutions

Bisphenol S (BPS) was procured from Global Lab Solutions (Delhi, India). Methylene blue was obtained from Nice Chemicals Private Limited (Kerala, India). N‐ω‐nitro‐L‐ arginine methyl ester (L‐NAME) was procured from Tokyo Chemicals Industry Co. Ltd (Tokyo, Japan) and atropine sulphate was purchased from Neon Laboratories Ltd (Maharastra, India). The stock solutions of all drugs were prepared in distilled water. BPS was dissolved in 100% ethanol to prepare a stock solution. Kreb Ringer solution was prepared according to the composition mentioned in Table 1.

**Table 1:** Composition of Kreb Ringer Solution

Salt	Concentration in mM
NaCl (sodium chloride)	137
KCl (potassium chloride)	2.68
CaCl_2._2H_2_O (calcium chloride-dihydrate)	1.8
MgCl_2_6H_2_O (magnesium chloride hexahydrate)	0.88
NaH_2_PO4.2H_2_O (sodium dihydrogen phosphate dihydrate)	0.36
NaHCO_3_ (sodium bicarbonate)	7
Glucose	11
pH	7.4

Experimental protocol

The experiments were divided into the following three groups: Group 1 (n=7), Group 2 (n-6), and Group 3 (n=15).

Group 1

Initially, the recording of baseline spontaneous contractions after stabilization was obtained. Thereafter, the right atrium was exposed to different concentrations of BPS (10^-6^-10mM) cumulatively. The atrial contractility was recorded for one minute at every five-minute interval for a total duration of 15 minutes after exposing the atrium to a particular concentration of BPS, before exposing it to the next higher concentration.

Group 2

After recording the baseline contractility, the tissue was exposed to various volumes of ethanol which were used to prepare different concentrations of BPS solution (10^-6^-10mM) and contractility was recorded as mentioned in Group 1. This group was examined to understand whether the changes in atrial contractility produced in Group 1 were due to BPS or the ethanol that was used to dissolve it.

Group 3

After obtaining the baseline recording, the atria was pre-treated with the following antagonists. This group was further subdivided into three subgroups.

Subgroup 1 (n=5): The right atria was pre-treated with atropine sulphate (0.3 µm) for 15 minutes followed by exposure to various concentrations of BPS (10^-6^-10mM) as mentioned earlier.

Subgroup 2 (n=5): The right atria was pre-treated with methylene blue for 15 minutes followed by exposure to various concentrations of BPS (10^-6^-10mM) as mentioned earlier.

Subgroup 3 (n=5): Right atrial tissue was pre-treated with L-NAME (10 µm) for 15 minutes followed by exposure to various concentrations of BPS (10^-6^-10mM) as mentioned earlier.

Statistical analysis

The rate and force of contractions were counted manually from the physiograph strip. The responses after exposure to a respective antagonist were taken as 100% in the pretreated groups. The mean ± standard error of the mean (SEM) of pooled data was calculated. One-way and two-way ANOVA followed by the Student-Newman-Keuls test for multiple comparisons were used to compare the concentration-response relationships with or without antagonists. A value of p < 0.05 was considered significant.

## Results

The initial rate of atrial contractions after stabilization was 127.43 ± 15.7 beats per minute (bpm) and the force of contraction was 7.84 ± 0.04 mN.

Decrease in rate of atrial contractions after exposure to BPS 

**Figure 1:**
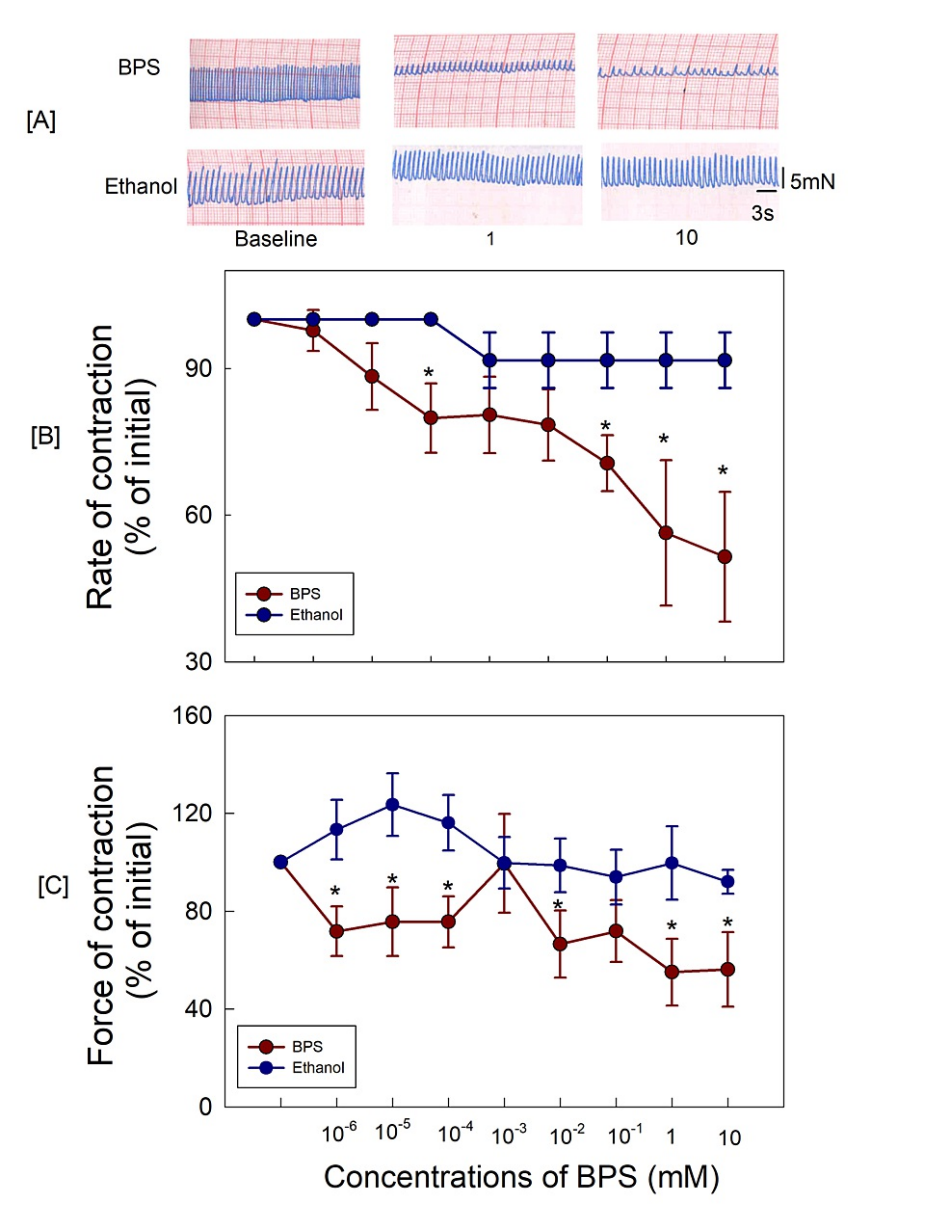
(A) The original recordings of BPS response at different concentrations and exposure of atria to ethanol. The original recordings of ethanol response at different concentrations show no significant decrease in rate and force of contraction of right atrial tissue; (B, C) Decrease in rate and force of contraction, respectively, caused by BPS. The responses in the ethanol-treated group were significantly different from the BPS-only group (p < 0.05, two‐way ANOVA). *p < 0.05 as compared with the BPS‐only group (Student–Newman–Keuls test for multiple comparisons) BPS: bisphenol S

Exposure to BPS (10^-6^-10 mM), decreased the rate of atrial contractions in a concentration-dependent manner. Maximal changes were observed after 15 minutes of exposure to BPS. The rate was reduced to 70.6% of the initial rate at 10^-1^ mM (p = 0.0002, one-way ANOVA), and exposure to 1mM BPS decreased the rate to 56.4% of the initial (p=0.0123, one-way ANOVA) and at 10 mM BPS, the rate further reduced to 51.5% of the initial value (p=0.003, one way ANOVA) (Figure 1).

Decrease in force of atrial contractions after exposure to BPS

Exposure to different concentrations of BPS decreased the force of atrial contractions (10^-6^-10 mM). At each concentration, maximal changes were observed at 15 min. The force decreased to 71.7% of the initial value after exposure to 10^-6^ mM BPS (p=0.034, one-way ANOVA) with a maximum decrease to 56.1% of initial force at 10 mM BPS (p=0.016, one-way ANOVA) (Figure 1).

BPS‐induced decrease in atrial contractions, effect of ethanol on right atrial tissue

To examine the effects of ethanol, which was used to dissolve BPS, the atria (n = 6) were exposed to ethanol (equi-volume) used to prepare different concentrations of BPS for the same duration as BPS. Ethanol did not show any significant changes in the rate and force of atrial contractions (Figure 1).

BPS‐induced changes in atrial contractions were not blocked by atropine sulphate

In this group (n=5), the right atria were pretreated with atropine sulphate (0.3 μM, 15 minutes), which is a muscarinic receptor blocker. On pre-treatment, atropine did not produce any significant change in the rate and force of atrial contractions (Table 2).

**Table 2:** Effect of various antagonists on rate and force of atrial contractions. The values are mean ± SEM from n = 5 observations for each L-NAME: N‐ω‐nitro‐L‐arginine methyl ester

Antagonist (µM)	Rate (beats/min)		Force (mN/gm atria)	
	Before	After	Before	After
Atropine (0.3)	113 ± 5.4	112 ± 6.7	9 ± 3.9	9 ± 2.1
Methylene Blue (100)	125 ± 5.0	98.5 ± 14.2	9.7 ± 2.7	13 ± 4.0
L-NAME (10)	122 ± 2.0	128.6 ± 6.4	7.2 ± 1.2	8.1 ± 1.8

**Figure 2:**
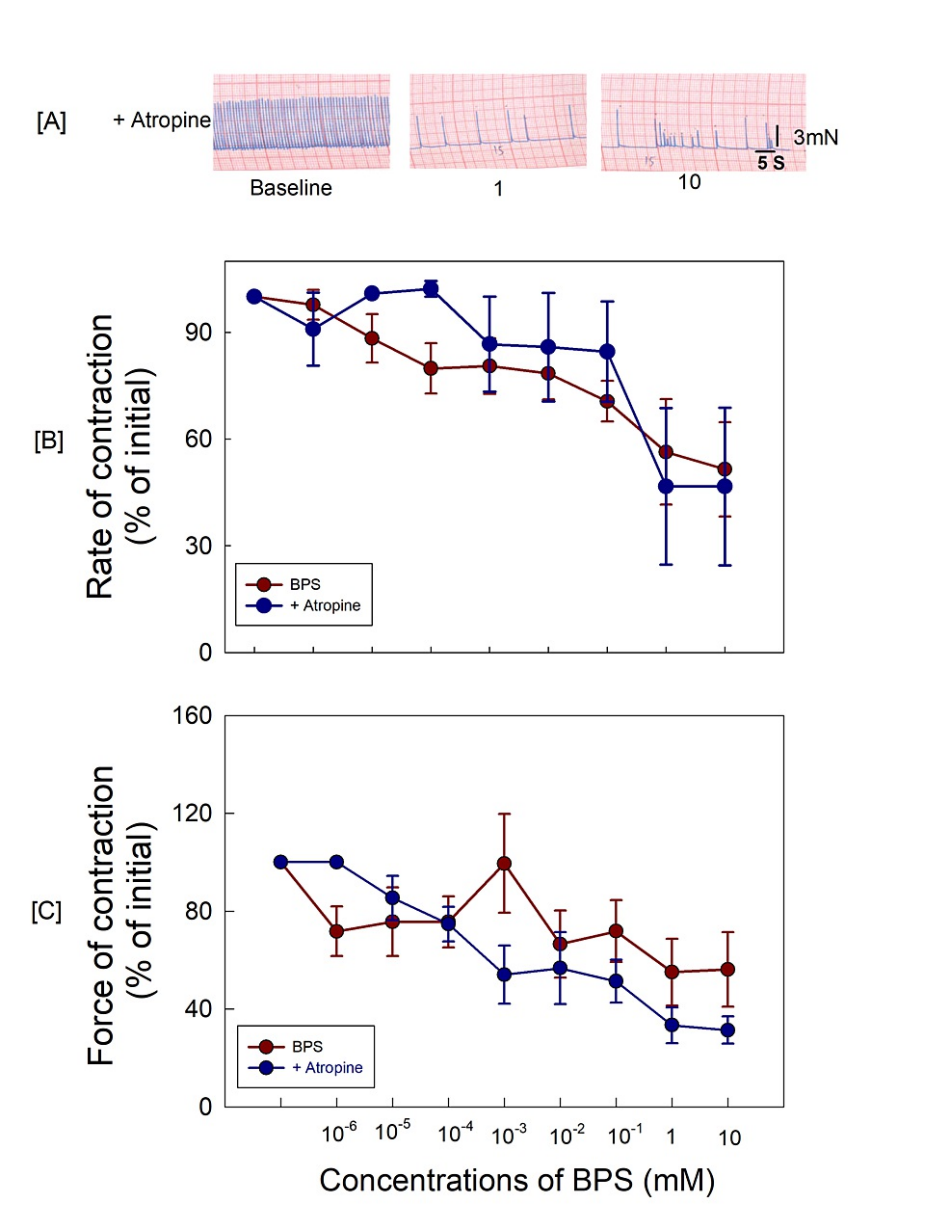
(A) The original recordings atrial contractions after 15 minute exposure to atropine sulphate; (B, C) Atropine could not block effects produced by BPS. BPS: bisphenol S

After pre-treating with atropine sulphate, the BPS‐induced decrease in rate and force of contractions was similar to the BPS‐alone treated group (Figure 2).

BPS-induced decrease in atrial contractions was blocked by L-NAME

**Figure 3:**
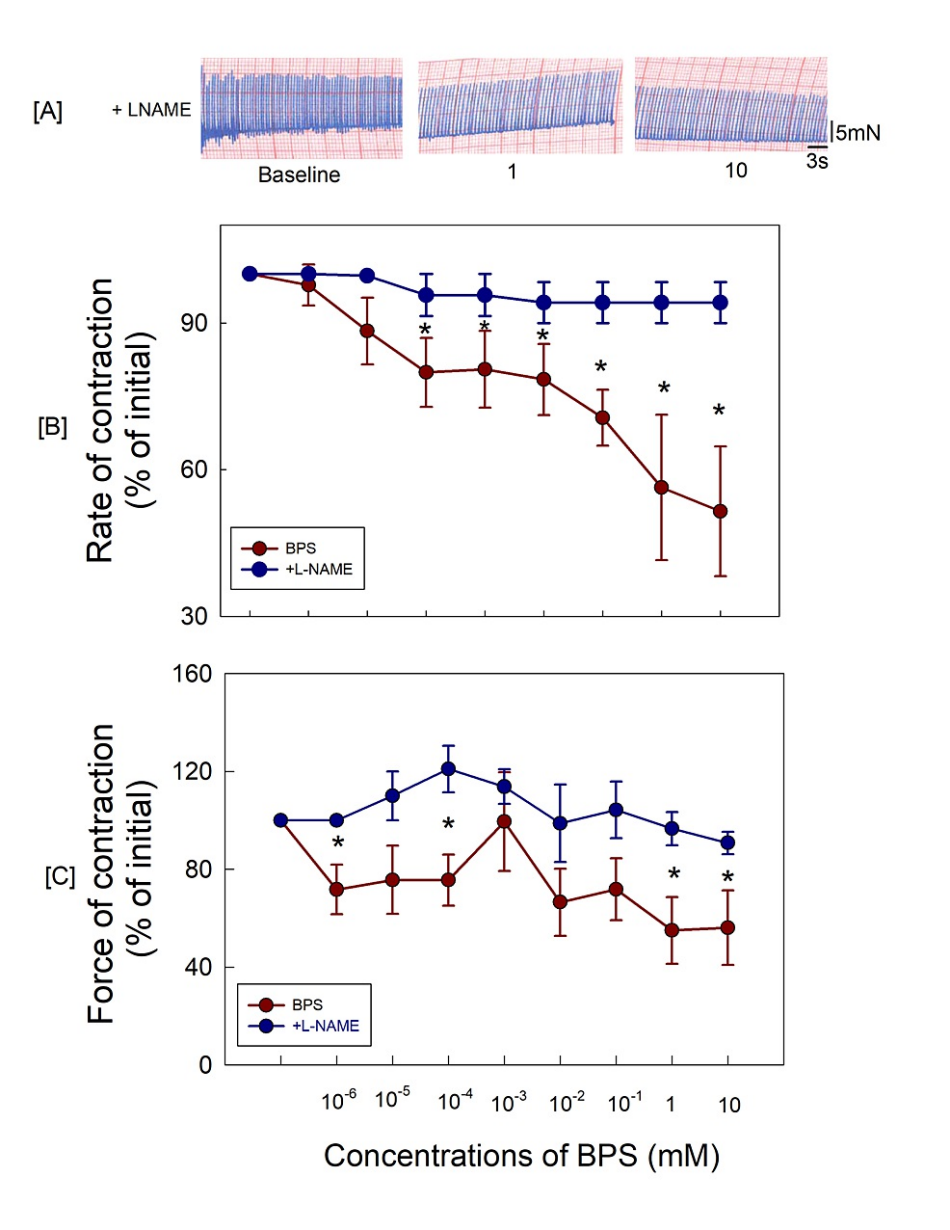
(A) The original recordings of atrial contractions after 15-minute exposure to L‐NAME; (B, C) Responses in the L‐NAME-treated group were significantly different from the BPS-only group (p < 0.05, two‐way ANOVA). *p < 0.05 as compared with the BPS‐only group (Student–Newman–Keuls test for multiple comparisons). L-NAME: N‐ω‐nitro‐L‐ arginine methyl ester; BPS: bisphenol S

The atria (n=5) were pretreated with L‐NAME (nitric oxide (NO) synthase inhibitor, 10 μM, 15 minutes) to examine the involvement of NO. The rate and force of atrial contractions were not affected significantly after pretreatment with L-NAME (Table 2). However, L‐NAME could block the BPS‐induced decreases in the rate and force of atrial contractions even on exposure to the highest dose of exposure to BPS (10 mM) (p<0.05, two-way ANOVA) (Figure 3).

BPS‐induced decrease in atrial contractions was not blocked by methylene blue

**Figure 4:**
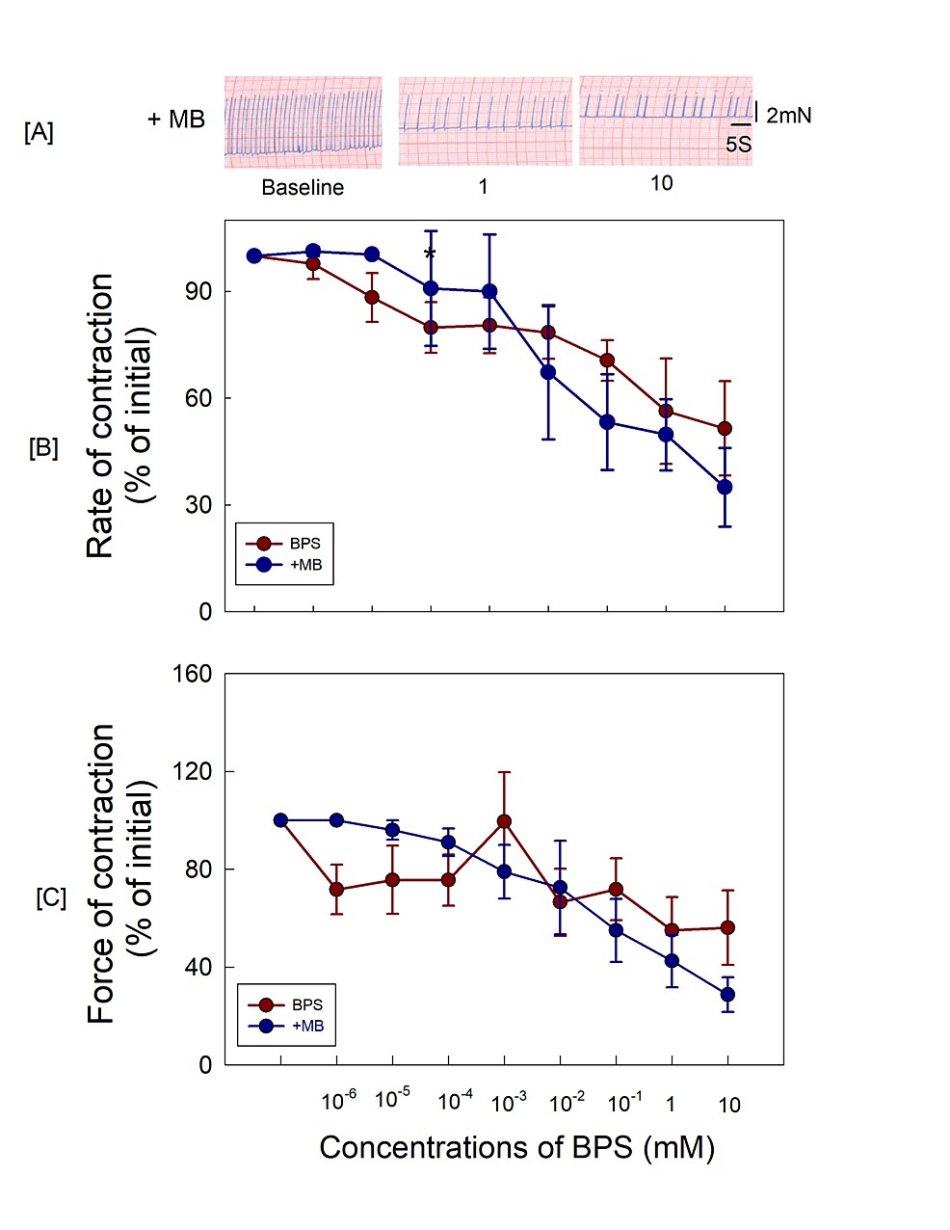
(A) The original recordings of atrial contractions after 15-minute exposure to MB; (B, C) MB could not block the BPS-induced changes in atrial contractility. MB: methylene blue; BPS: bisphenol S

Pretreatment with methylene blue (a guanylyl cyclase inhibitor, 100 μM, 15 minutes) per se did not affect the atrial contractility significantly (Table 1). The BPS‐induced changes in atrial contractility could not be blocked by methylene blue (Figure 4).

.

## Discussion

The observations in the present study demonstrated that BPS depressed the in vitro spontaneous contractions of the right atria. Further, the observations from the present study revealed that the BPS-induced decrease in atrial contractility was due to the involvement of the NO pathway. BPS decreased the rate and force of spontaneous contractions of the right atria indicating the direct effect of BPS on atrial contractility. BPS was dissolved in ethanol and it decreased the rate and force of right atrial contractions; hence, it was necessary to rule out that the changes in the contractility of right atria were due to BPS alone and not ethanol. Hence, in a separate set of experiments, right atria were exposed to ethanol in the same concentrations used to dissolve BPS to prepare various concentrations/ dilutions of BPS (10^-6^-10mM). The results demonstrated that the depressant action of BPS is not attributed to ethanol but due to BPS alone.

The mechanism of toxicity produced by BPS may be expected due to the activation of the cholinergic system, which can lead to a decrease in the rate and force of contractions of the right atria. In studies reported elsewhere, *Ptychodiscus brevis* (PbTx) toxin and *Mesobuthus tamulus* (MBT) venom were found to decrease cardiac contractility due to the activation of cholinergic receptors (muscarinic) [24,25]. However, in the present study, atropine could not block the BPS-induced changes in atrial contractility and therefore the effects produced by BPS could not be attributed to activation of cholinergic receptors. In a previous study by Pant et al., BPA was reported to decrease right atrial contractility and BPA did not act through cholinergic receptors [26].

BPS is an analogue of BPA and the mechanism of action on right atrial contractions is expected to be similar to BPA. Therefore, the involvement of NO may be a possibility as demonstrated for BPA by Pant et al. [26]. In the presence of enzyme NO synthase (eNOS), NO is produced from arginine in the endothelial cells, endocardial cells, ventricular myocytes and other myocardial cells [27,28]. The NO produced from the reaction enters cardiac myocytes and affects cardiac contractility depending upon NO concentrations [27]. NO influences cardiac contractility in a concentration-dependent biphasic manner. It is reported that NO at higher concentration decreases the cardiac contractility and at lower levels increases the contractility. In the present study, BPS reduced right atrial contractility and these changes were blocked by L-NAME, a NO synthase inhibitor. Therefore, BPS is expected to work through increasing NO levels in cardiac myocytes. It is also reported that at higher concentrations of NO, soluble guanylyl cyclase (sGC) may be activated [27]. However, in the present study, BPS‐induced decreases in atrial contractility were not prevented by pretreatment with methylene blue (a guanylyl cyclase inhibitor). Hence, BPS is expected to produce a depressive effect on atrial contractility through NO-cGMP-independent mechanisms, unlike BPA which is reported to decrease atrial contractility involving the NO‐dependent G‐cyclase signalling pathway as reported by Pant et al. [26]. Oxidation or trans-nitrosylation of NO may influence several ion channels in cardiac cells. It is reported to decrease sodium influx in cardiomyocytes through activation of protein kinase A and protein kinase G pathways. It may also reduce calcium influx through cGMP-independent mechanisms [29]. Inhibition of inward calcium current is also reported [30]. Therefore, BPS seems to affect atrial contractility through the direct action of NO influencing the cardiac ion channels in this case. However, the evaluation of mechanisms affecting the ionic channels was beyond the scope of the present study and needs to be examined further.

## Conclusions

In the present study, BPS produced a significant decrease in right atrial rate and force of contraction. Further, the present study provides evidence for the involvement of NO-cGMP-independent pathways in BPS‐induced cardiotoxicity. It is pertinent to understand that BPS, although presently considered a safe alternative to BPA, possesses toxic effects even in low concentrations. Therefore, more studies are required to explore the chemical before tagging it safe for human usage.
